# Characterization of a novel non-steroidal glucocorticoid receptor agonist optimized for topical treatment

**DOI:** 10.1038/s41598-022-05471-w

**Published:** 2022-01-27

**Authors:** Stefan Eirefelt, Martin Stahlhut, Naila Svitacheva, Martin A. Carnerup, Joel Mauricio Correa Da Rosa, David Adrian Ewald, Troels T. Marstrand, Mikkel Krogh-Madsen, Georg Dünstl, Kevin Neil Dack, Anna Ollerstam, Hanne Norsgaard

**Affiliations:** LEO Pharma A/S, Industriparken 55, Ballerup, Denmark

**Keywords:** Drug discovery, Medical research

## Abstract

Glucocorticoids (GCs) are commonly used topical treatments for skin diseases but are associated with both local and systemic side effects. In this study, we describe a selective non-steroidal glucocorticoid receptor (GR) agonist for topical use, LEO 134310, which is rapidly deactivated in the blood resulting in low systemic exposure and a higher therapeutic index in the TPA-induced skin inflammation mouse model compared with betamethasone valerate (BMV) and clobetasol propionate (CP). Selectivity of LEO 134310 for GR was confirmed within a panel of nuclear receptors, including the mineralocorticoid receptor (MR), which has been associated with induction of skin atrophy. Topical treatment with LEO 134310 in minipigs did not result in any significant reduction in epidermal thickness in contrast to significant epidermal thinning induced by treatment with BMV and CP. Thus, the profile of LEO 134310 may potentially provide an effective and safer treatment option for skin diseases compared with currently used glucocorticoids.

## Introduction

Topical glucocorticoids (GCs), also referred to as topical corticosteroids (TCS) are efficacious and widely used drugs in dermatology due to their broad anti-inflammatory activity but are associated with adverse effects preventing long-term clinical use. In particular, development of skin atrophy is an important limiting factor for the use of TCS.

The mechanism of action of the glucocorticoid receptor (GR) has been extensively studied. Upon binding of GC to GR, the GC/GR complex translocates to the nucleus where it acts as a transcription factor to regulate a multitude of target genes. The activated GR can mediate transactivation and transrepression, i.e., activation or repression of gene transcription, respectively. Regulation of target gene transcription may occur via direct binding to glucocorticoid response elements (GREs) on the DNA or by binding of GR to other transcription factors. It can also involve interaction with various co-activators and co-repressors. One of the most well-described activities of GCs is the inhibition of pro-inflammatory gene transcription via inhibition of NF-κB and AP-1 activity^1^.

Generally, anti-inflammatory activity of GCs was perceived to be associated with transrepression and adverse effects attributed to transactivation. This concept was driving the development of GR ligands with dissociated profiles, predominantly favouring transrepression activity. However, this concept turned out to be oversimplified illustrated by limited anti-inflammatory efficacy of dissociated GR ligands in mouse models or in clinical trials. Indeed, anti-inflammatory activity is not associated exclusively with transrepression and neither are adverse effects with transactivation^2^.

GCs do not only bind to GR but can also bind to the structurally related mineralocorticoid receptor (MR) and induce gene transcription through binding to GREs^3^. Epidermal overexpression of either GR or MR in mice revealed a highly similar skin phenotype, including disturbed epidermal differentiation and skin atrophy^4,5^. Furthermore, mice with an epidermis-specific deletion of MR had increased keratinocyte proliferation and differentiation and showed a partial reversal of dexamethasone-induced epidermal thinning^6^. Interestingly, pharmacological modulation of MR activation by treatment with MR antagonists limited clobetasol propionate (CP)-induced skin atrophy both in human skin explants and in a small clinical study in healthy volunteers^7^. Thus, skin atrophy may be limited for a GR agonist with high selectivity for GR over MR.

A systemic adverse effect associated with long-term systemic exposure to GCs is suppression of the hypothalamic–pituitary–adrenal (HPA) axis. From a meta-analysis of 12 clinical trials, high potency TCS (class 1) have been shown to suppress the HPA axis in children with AD^8^. Furthermore, potent TCS such as betamethasone dipropionate and CP have been shown to reduce morning plasma cortisol significantly^9^. However, cortisol decrease only results in pathological HPA axis suppression in very few cases^10^. Another adverse effect observed after prolonged systemic treatment with GCs is osteoporosis^11,12^. Long-term systemic exposure to oral GCs is associated with reduced bone mineral density and increased risk for fractures^13,14^.

Here we report on the preclinical characterization of LEO 134310, a non-steroidal, potent and selective GR agonist. LEO 134310 retained full efficacy on GR transactivation and transrepression. To minimize systemic exposure, LEO 134310 was designed as a “dual-soft” drug rapidly deactivated in the blood and other compartments by enzymatic hydrolysis^15–17^. Due to its reduced potential for skin atrophy and low systemic exposure, LEO 134310 may be suitable for long-term topical treatment of inflammatory skin diseases.

## Results

### LEO 134310 displays rapid turn-over in blood and high in vivo clearance

LEO 134310 is a non-steroidal GR agonist consisting of a gamma-hydroxy-butyrolactone derivatized with a mixed aromatic/aliphatic substituent containing a central benzoate ester and two amides^15^. The lactone bond constitutes a dominant soft spot for enzymatic hydrolysis leading to a pronounced loss of activity following ring opening. The corresponding major metabolite is designated as LEO 134998. The calculated properties and compound structures of LEO 134310 and LEO 134998 together with CP and BMV can be seen in Table 1.

**Table 1:** Structures and calculated properties of LEO 134310, metabolite (LEO 134998) and reference GCs Clobetasol Propionate (CP) and Betamethasone Valerate (BMV). *H acceptors* hydrogen bond acceptors, *H donors* hydrogen bond donors, *PSA* polar surface area.

Compound	MW (g/mol)	PSA	H acceptors	H donors	Structure
CP	467	81	5	1	
BMV	477	101	6	2	
LEO 134310	605	120	8	1	
LEO 134998	645	215	9	2	

LEO 134310 is rapidly metabolized in vitro in whole blood and hepatocytes from different species and has a high clearance in vivo in rat and dog (5- and 15-fold the liver blood flow, respectively) as shown in Table 2.

**Table 2:** Metabolic in vitro stability of LEO 134310 in mouse, rat, dog, minipig and human whole blood and hepatocytes and in vivo PK parameters in rat and dog. Predicted hepatic clearance (CL_*H*_) from hepatocytes (well stirred model). Mean values ± S.D.

Parameter	Rat	Dog	Minipig	Human
In vitro (blood) half-life (min)	2.2 (2.1, 2.2) (n = 2)	3.3 ± 2.1 (n = 5)	3.8 (n = 1)	4.4 ± 2.2 (n = 4)
In vitro (hepatocytes) CL_*H*_ (mL/min/kg)	57 (n = 1)	> 30 (n = 1)	> 35 (n = 1)	> 18 (n = 1)
In vivo clearance (mL/min/kg)	360 ± 85 (n = 3)	470 ± 97 (n = 3)	–	–
In vivo terminal half-life (min)	190 ± 171 (n = 3)	11 ± 1.2 (n = 3)	–	–

The predicted clearance of LEO 134310 in man is 300 mL/min/kg (15-fold the liver blood flow) based on allometric scaling of clearance in dogs, using 0.75 as scaling factor. The corresponding predicted half-life is < 5 min, which is in line with the measured in vitro half-life of LEO 134310 in human blood (4.4 min) (Table 2).

### LEO 134310 is a potent and selective GR agonist

There are known GR agonists in development that utilize non-steroidal structural motifs, such as indazoles. In contrast, LEO 134310 was designed by identifying a new core template that consists of a 4-(2-amido-1-aryl-propyloxy)-benzoate ester with good GR agonist activity, and then appending a lactone substituent in a position that both increased GR agonism and also provided a site for rapid metabolism in whole blood to give a less active hydroxy acid^15^.

In a GR binding assay, LEO 134310 showed high affinity (EC_50_ of 14 nM). Furthermore, LEO 134310 achieved full efficacy (E_max_ of 100 and 95%, respectively) in transrepression and transactivation assays using HeLa reporter cell lines (Table 3). The agonist potency of LEO 134310 was 1.2 nM on transrepression which was comparable to the potency of BMV (0.57 nM). Transactivation by LEO 134310 showed an EC_50_ of 410 nM and thus more than 300-fold higher concentration than needed for transrepression. This is in contrast to CP and BMV that both showed sub-to-low nanomolar potencies in either assay. LEO 134998, the major metabolite of LEO 134310, lost about 40-fold potency in the transrepression assay compared to LEO 134310. Transactivation potency was comparable to the parent compound LEO 134310 at 560 nM.

**Table 3:** In vitro potency of LEO 134310 and comparison to CP and BMV. GR agonist-induced transactivation of mouse mammary tumour virus (MMTV) promoter in HeLa cells and PMA-induced, GR-mediated transrepression of human Matrix Metallopeptidase 1 (MMP1) promoter in HeLa cells of LEO 134310 (n = 2), its major metabolite, LEO 134998 (n = 2), CP (n = 6–7) and BMV (n = 1–2). Inhibition of LPS-induced TNF-alpha release in PBMCs^a^ isolated from Human, Mouse, and Pig whole blood. LEO 134310 (n = 15, 2, 2), its major metabolite, LEO 134998 (n = 10, 3, 5), CP (n = 17, 4, 4) and BMV (n = 15, 2, 2). Geometric mean (95% confidence intervals within brackets for n > 2. For n = 2 individual results are shown. *p < 0.05 compared to LEO 134310.

Compound	Transactivation EC_50_ (nM) E_max_ (%)	Transrepression EC_50_ (nM) E_max_ (%)	Human^a^ EC_50_ (nM) E_max_ (%)	Mouse^a^ EC_50_ (nM) E_max_ (%)	Pig^a^ EC_50_ (nM) E_max_ (%)
CP	0.25 (0.078–0.83) 110	0.064 (0.029–0.15) 90	0.52 (0.22–1.2) 79	0.15 (0.053–0.43) 95	0.38 (0.27–0.54) 95
BMV	2.6 (n = 1) 120	0.57 (0.40, 0.82) 78	3.2 (1.5–6.7) 79	0.22 (0.18, 0.27) 95	1.5 (1.2, 1.8) 99
LEO 134310	410 (340, 480) 95	1.2 (1.2, 1.3) 100	2.3 (0.61–8.4) 77	0.58 (0.25, 1.3) 102	3.2 (1.4–7.2) 96
LEO 134998	560 (520, 590) 88	46* (41, 50) 98	260* (77–902) 75	35* (22–58) 100	210* (140–330) 93

Inhibition of LPS-induced TNF-alpha release by LEO 134310 in a human PBMC assay was observed at low nanomolar potency (Table 3). CP was fourfold more potent compared to LEO 134310 while BMV was equipotent to LEO 134310. Similarly, TNF-alpha inhibition assays were employed with mouse and pig PBMCs. All compounds were slightly more potent in mouse PBMCs and LEO 134310 was slightly less potent than CP and BMV in both species, yet still reducing TNF-alpha levels in the low nanomolar range (Table 3). The metabolite LEO 134998 was 60- and 100-fold less potent in the three species. In summary, LEO 134310 behaved as a full GR agonist with high potency in relevant inflammatory functional assays.

**Figure 1:**
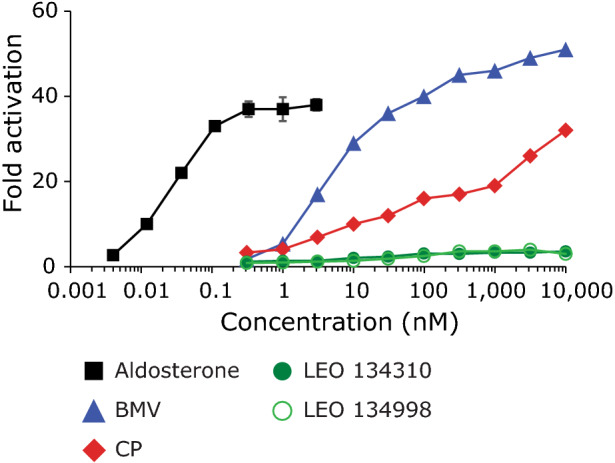
LEO 134310 does not induce mineralocorticoid receptor (MR) signalling. Dose–response curves for induction of luciferase activity in MR-luciferase reporter cells by aldosterone, CP, BMV, LEO 134310, and LEO 134998 after incubation for 24 h.

Selectivity of LEO 134310 for MR was investigated in agonist and antagonist assays on MR. No significant activation of MR was observed in a specific reporter cell line, in contrast to the positive control aldosterone and two steroidal GR agonists, CP and BMV (Fig. 1). The metabolite LEO 134998 did not activate MR either. Furthermore, no antagonist activity on MR was observed for LEO 134310 or LEO 134998 in the MR reporter assay (Supplementary Fig. S1). In addition, no significant interaction of LEO 134310 or LEO 134998 with nine different nuclear hormone receptors (PPAR-alpha, LXR-beta, RAR-alpha, RXR-alpha, ER-alpha, PR, AR, ER-beta and TR) was detected in specific binding assays (Supplementary Fig. S2). Moreover, both compounds were tested at 10 µM in a panel of 8 ion channels (Eurofins IonChannelProfiler), 40 kinases, 2 phosphatases (Eurofins KinaseProfiler), and up to 163 G-protein coupled receptors (Eurofins GPCRProfiler) and no off-target activity was identified. Like for BMV and CP, inhibition of LPS-induced cytokine release by LEO 134310 in a human PBMC assay could be reversed in a concentration-dependent manner by the steroidal GR antagonist RU486 (Supplementary Fig. S3). Thus, LEO 134310 is highly selective for GR.

### Effective topical delivery and pharmacological activity of LEO 134310 in human skin

With the purpose to investigate the pharmacological activity of LEO 134310 in human skin after topical dosing, propylene carbonate formulations of LEO 134310, CP and BMV were applied on human skin explants for 24 h and compared to vehicle controls. The dermal concentrations were measured and found to be proportional to dose for all three compounds. For LEO 134310, higher doses were needed to achieve similar dermis concentrations compared to CP (7-fold) and BMV (3-fold). Dermis concentrations of the major metabolite, LEO 134988, were comparable to parent indicating hydrolysis in epidermis and dermis (Supplementary Fig. S4).

GR target engagement in the skin was measured by mRNA levels of FK506 binding protein 5 (*FKBP5*), a gene directly induced by GR transactivation. Topical dosing of LEO 134310 resulted in full agonism with a maximal effect comparable to CP and BMV (Table 4). Based on the relative gene induction of *FKBP5*, an approximately 30-fold higher dose of LEO 134310 was needed to achieve similar level of activity as BMV. However, this may be a conservative estimate as the readout is based on a transactivation response where LEO 134310 showed notable lower potency relative to transrepression. Full agonism and potency of LEO 134310 was supported by additional gene expression of glucocorticoid-induced leucine zipper (*GILZ*) and thioredoxin-interacting protein (*TXNIP*), directly induced by GR transactivation (Supplementary Table S1).

**Table 4:** Pharmacological activity of LEO 134310A vs CP and BMV in human skin assessed by gene expression levels of *FKBP5*. Effective doses (API concentrations in dosing solution) of CP (n = 6 studies), BMV (n = 3 studies) and LEO 134310A (n = 5 studies) to induce *FKBP5* levels to 50% of maximum response (ED_50_). *FKBP5* levels were normalised to vehicle-treated controls and E_max_ and ED_50_ were estimated in a sigmoid E_max_ model. Weighted geometric mean ± standard deviation. Statistical comparison (T-test) against BMV (*NS* not significant).

Compound	Relative ED_50_ (µg/mL)	E_max_ (fold to control)
CP	0.52 ± 0.06^NS^	6.9 ± 3.1^NS^
BMV	9.5 ± 6.9	5.6 ± 4.9
LEO 134310	270 ± 130**	7.2 ± 2.7^NS^

### Lack of skin atrophy induction by LEO 134310 in minipigs

With the aim to assess local tolerance, 5 minipigs were topically dosed with LEO 134310 (2%), CP (0.05%) and BMV (0.1%) for 4 weeks under semi-occlusion for 6 h daily. To take into account possible effects of different vehicles, CP and BMV were tested by use of the marketed products, Dermovate and Betnovate, and additionally by formulations in the same vehicle as LEO 134310.

**Figure 2:**
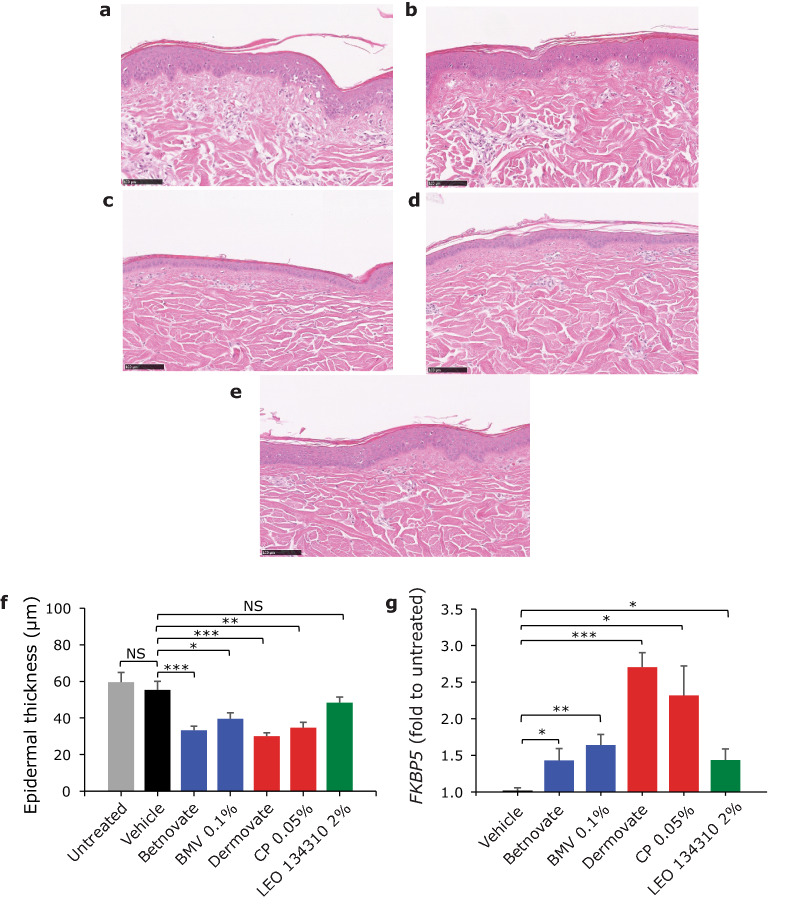
LEO 134310 does not induce skin atrophy in minipigs. H&E staining of minipig skin biopsy samples from untreated skin (**a**), test fields treated with LEO vehicle (**b**), Dermovate cream 0.05% CP (**c**), Betnovate cream 0.1% BMV (**d**), and LEO 134310 2% (**e**). The bar represents 100 µm. Epidermal thickness in skin biopsies from minipigs after 4 weeks treatment (**f**) (n = 5). Statistical significance was determined by one-way ANOVA with Dunnett’s multiple comparisons test versus vehicle. *p < 0.05; **p < 0.01; ***p < 0.001; NS: not significant. Fold changes of *FKBP5* expression in relation untreated control, measured by qRT-PCR in skin biopsies from minipigs treated for 4 weeks (**g**) (n = 5). The differences were modelled in a paired manner applying a linear mixed effects model, with Dunnett’s post hoc testing (*p < 0.05; **p < 0.01; ***p < 0.001). Error bars represent standard errors of the paired differences.

In contrast to treatment with LEO 134310, notable discoloration was observed in most application sites treated with CP and BMV formulations. Compared to untreated and vehicle-treated controls, a pronounced and statistically significant reduction in epidermal thickness (30–40%) was found after treatment with CP and BMV, independent of the formulation. Treatment with LEO 134310 did not result in any significant reduction in epidermal thickness compared to untreated and vehicle-treated controls (Fig. 2).

To assess the pharmacological activity of the compounds in the skin after topical dosing, *FKBP5* was used as GR target engagement marker. Treatment with LEO 134310 and BMV induced similar levels of *FKBP5* gene expression whereas higher levels of *FKBP5* gene expression were induced by CP (Fig. 2). Thus, at similar levels of induced GR activity BMV showed pronounced skin atrophy in contrast to LEO 134310.

### Improved therapeutic index of LEO 134310 compared to CP and BMV in TPA mouse model

**Figure 3:**
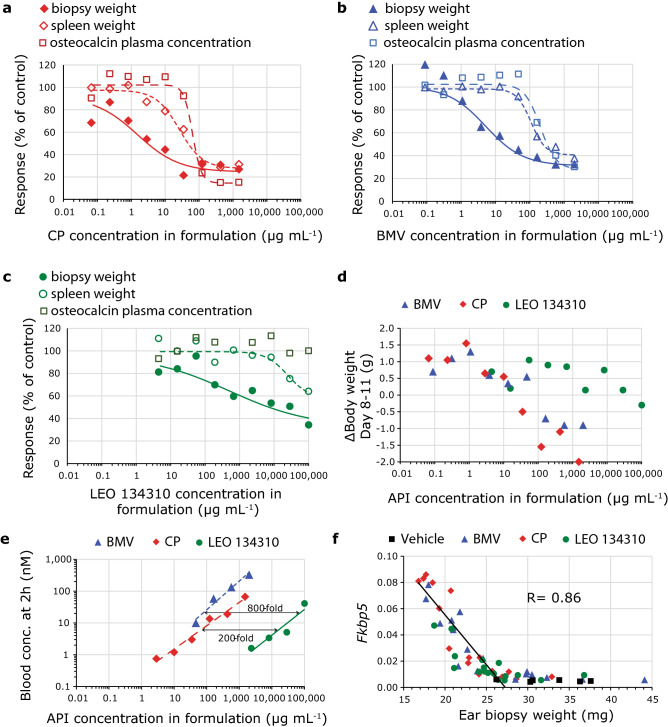
In vivo efficacy versus systemic side effects of LEO 134310, CP and BMV in the TPA-induced skin inflammation mouse model. Dose–response curves for effect of CP (**a**), BMV (**b**) and LEO 134310 (**c**) on ear biopsy weight, spleen weight and osteocalcin plasma concentration in the TPA mouse model. All values are normalized to vehicle/TPA controls. Body weight vs. dose (**d**), blood concentration at 2 h after dosing vs. dose (**e**) and mRNA levels of *Fkbp5* vs ear biopsy weight (**f**) (R = correlation coefficient). Vehicle, (**a**) CP, (**b**) BMV, (**c**) LEO 134310. Mean values at each dose (n = 2) in (**a**–**e**) and individual values in (**f**).

To investigate in vivo efficacy and the impact of the high clearance, LEO 134310 was tested head-to-head against CP and BMV in the TPA-induced skin inflammation model in mouse^18^. This model allows evaluation of efficacy together with early detection of systemic side effects. The doses of LEO 134310 needed to achieve systemic exposure (blood concentrations at 2 h after the last topical dose) comparable to CP and BMV were 200- and 800-fold higher, respectively (Fig. 3e).

A dose-dependent improvement of inflammation measured as reduction in ear biopsy weight was observed for all three compounds (Fig. 3a–c). LEO 134310 was approximately 300- and 100-fold less potent than CP and BMV, respectively (Table 5). Furthermore, a dose-dependent increase in mRNA levels of *Fkbp5* and *Gilz* was observed for all compounds. *Fkbp5* and *Gilz* mRNA levels correlated inversely with ear biopsy weight, associating levels of GR activity with the anti-inflammatory effect (Fig. 3f and Supplementary Fig. S5). A dose-dependent reduction of body weight gain, spleen weight, and plasma osteocalcin levels, indicating systemic exposure, was observed for CP and BMV but not—or only to a lesser extent—for LEO 134310 (Fig. 3a–d). Since ID_50_ values could not be determined for the reduction of spleen weight or osteocalcin concentrations induced by LEO 134310, the values provided in Table 5 were extrapolated and thus carry some uncertainty. A 2-fold higher therapeutic index (TI) to ear weight ID_50_, compared to the spleen weight ID_50_ and a > 5-fold higher TI, compared to the osteocalcin ID_50_, was estimated for LEO 134310 compared to CP and BMV (Table 5).

**Table 5:** Higher therapeutic index for LEO 134310 vs CP and BMV in the TPA-induced skin inflammation mouse model. Calculated inhibitory doses (API concentration in formulation) to reach 50% of I_max_ effect on ear biopsy weight, spleen weight and osteocalcin concentration (ID_50_) and estimated therapeutic index (TI). CV (%) within brackets. ^a^Therapeutic index (Spleen ID50/Ear ID50). ^b^Therapeutic index (Osteocalcin ID50/Ear ID50).

Compound	Ear biopsy weight		Spleen weight			Osteocalcin concentration		
	ID_50_ (µg/mL)	I_max_ (%)	ID_50_ (µg/mL)	I_max_ (%)	Therapeutic index^a^	ID_50_ (µg/mL)	I_max_ (%)	Therapeutic index^b^
CP	1.5 (69)	67 (14)	26 (27)	70 (8)	17	64 (17)	88 (4)	43
BMV	5.1 (73)	69 (17)	110 (25)	58 (9)	21	200 (24)	74 (10)	39
LEO 134310	500 (250)	64 (39)	25,000 (110)	42 (56)	49	–	–	> 200

## Discussion

We have demonstrated in vitro and in vivo efficacy of LEO 134310, a novel non-steroidal GR agonist. LEO 134310 is a full GR agonist with high potency on transrepression and lower potency on transactivation. This profile is different from dissociated GR agonists designed to possess partial transactivation activity on GR^19–21^. High selectivity of LEO 134310 for GR was observed, as no interactions with nine common nuclear hormone receptors could be detected. As MR activation has been associated with development of skin atrophy^5^, agonist and antagonist activities of LEO 134310 on this receptor were analyzed in greater detail. In vitro data demonstrated that in contrast to BMV and CP, LEO134310 did not activate MR. In line with this result, low atrophogenic potential of LEO 134310 was demonstrated in a minipig study, supporting that lack of MR activation may indeed be a critical feature of LEO 134310.

In human skin explants, full GR agonism was observed for LEO 134310 as for BMV and CP but at considerably higher doses. The dissociating GR agonist, GW870086, with selectivity against MR and minimal transactivation activity^21^, has been reported not to induce skin atrophy in healthy subjects. However, GW870086 did not achieve significant clinical efficacy in atopic dermatitis patients^22^.

The different potencies observed for CP, BMV and LEO 134310 in human skin explants could be due to differences in skin penetration and stability in the skin compartment. It has previously been demonstrated that total percutaneous absorption and absorption rate correlate inversely with molecular weight (MW) and the number of hydrogen bond acceptor (HA) groups^23–25^. These parameters were higher for LEO 134310 and may possibly contribute to lower dermis concentrations of LEO 134310 compared to CP and BMV. Regarding dermal stability, similar levels of exposure were observed for LEO 134310 and its inactive metabolite LEO 134998, indicating significant hydrolysis in this compartment. In addition, inherent potency and tissue binding in the dermis could contribute to the lower potency of LEO 134310 compared with CP and BMV in human skin explants.

In the TPA mouse model the maximal anti-inflammatory effect was comparable for CP, BMV and LEO 134310, respectively, although higher doses of LEO134310 were required for an equivalent effect. Similar to the human skin explant model, the higher doses needed for equivalent anti-inflammatory effect in vivo could be explained by different skin penetration, stability, in vitro potency and skin binding. In addition, systemic exposure of active parent compound contributes substantially to the anti-inflammatory effect of TCS in small animals. This was demonstrated for CP in a separate TPA study in mice where CP was applied topically on the non-inflamed ear (tested dose range 0.03–3 µg/ear). Significant anti-inflammatory effect due to systemic exposure was seen from 1 µg/ear (Supplementary Fig. S6).

LEO 134310 was designed to be cleared rapidly upon entering the systemic circulation. The half-life in human whole blood was short (6 min) and the predicted hepatic clearance was high (> 18 ml/min/kg). The systemic clearance was predicted by allometric scaling of clearance in dog. It has been demonstrated that esterase activity and hydrolysis pattern in dog and human plasma is very similar^26^. In addition, clearance in man was well predicted by allometric scaling of clearance in dog, for a number of ester drugs^27–38^. LEO 134310 was predicted to have a systemic clearance that is more than 10-fold higher than the liver blood flow in man. This indicates that clearance is largely driven by extrahepatic components. The corresponding predicted half-life in man was less than 5 min which fits well with the in vitro half-life in blood.

Based on these data, LEO134310 may pose a lower risk of systemic target-related side effects due to the rapid inactivation by hydrolysis upon entry into the circulation. For more stable potent GR agonists, systemic exposure after long-term topical application poses some risk for systemic adverse effects^8,9^. We have shown in the mouse TPA model that LEO 134310 did not suppress osteocalcin levels at efficacious doses on skin inflammation, while BMV and CP did. In addition, body weight and spleen weight were affected to a lower degree by LEO134310 compared to BMV and CP. Our data suggest that the efficacy of LEO 134310 may be in the range of a US class 3 potency (e.g. BMV), but with a lower atrophogenicity. Safety, tolerability and pharmacodynamic effects of LEO 134310 were evaluated in a Phase 1b study in adults with chronic plaque psoriasis (NCT03669757; data not yet published).

## Methods

### Compounds and reagents

Acetone, TPA (12-*O*-tetradecanoylphorbol-13-acetate), CP and BMV were purchased from Sigma Aldrich (DK). Transcutol P was from Gattefosse (FR). LEO 134310 and LEO 134998 were synthesized at LEO Pharma. Dermovate cream (0.05% CP; GSK) and Betnovate cream (0.1% BMV; Oriform) were purchased at the local pharmacy.

### In vitro potency studies

#### GR binding assay

Binding of GR agonists to human GR was evaluated in a fluorescence polarization assay. 2.5 nM Fluormone GS1 (Thermo Fisher) and 4 nM recombinant human full-length GR (Thermo Fisher) were incubated in the presence of individual test and control compounds. Changes in polarization values were used to determine the relative affinity of test compounds for the GR.

#### TNF-alpha inhibition assays

PMBCs were isolated from human buffy coats or animal whole blood (EDTA-treated). Buffy coats from anonymized healthy adult blood donors were obtained from the Blood Bank of the National University Hospital Copenhagen in accordance with national legislation. Whole blood from mice (obtained from Charles River) was sampled at the animal facility at LEO Pharma and whole blood from pigs was sourced from ScanTox. Test compounds and PBMCs were incubated with 1 µg/ml LPS (Sigma) overnight at 37 °C in HEPES and Glutamax-containing RPMI medium supplemented with either 0.5% human serum albumin (human PBMCs) or 10% fetal bovine serum (mouse and pig PBMCs) and penicillin/streptomycin. TNF-alpha was measured using AlphaLISA (Perkin Elmer).

#### Transrepression and transactivation assays

Assays were adopted from a method reported by Schäcke et al.^39^ and HeLa cells were obtained from the American Type Culture Collection (ATCC). Transrepression assay: A HeLa cell line stably expressing luciferase under the control of the human Matrix Metallopeptidase 1 (MMP1) promoter was generated. Upon binding to the GR, GR agonists can bind to AP-1, preventing it from binding the MMP1 promoter, resulting in inhibition of luciferase transcription and activity. Briefly, cells were incubated in culture medium containing charcoal-filtered fetal bovine serum with 5 nM PMA (phorbol 12-myristate 13-acetate) for 18 h with or without test compounds. Then, luciferase activity was detected luminometrically after addition of specific substrate to the cells.

#### Transactivation assay

A HeLa cell line stably expressing luciferase under the control of the mouse mammary tumour virus (MMTV) promoter was generated. When the GR is activated by addition of a GR agonist, it forms a homodimer and binds to the MMTV promoter, t inducing transcription of the luciferase reporter gene. Briefly, cells were incubated with test compounds in culture medium containing charcoal-filtered fetal bovine serum for 20 h 37 °C 5% CO_2_. Finally, luciferase activity was detected luminometrically after addition of specific substrate to the cells.

#### Pharmacological analysis of in vitro assays

IC_50_ of test compounds was determined by 10-point titrations in duplicates. IC_50_ values and other parameters were calculated by sigmoidal curve fitting in Assay Explorer.

#### MR agonist and antagonist assays

Assays were performed at Indigo Biosciences according to standard protocols. In brief, MR-luciferase reporter cells were incubated with medium containing charcoal-stripped fetal bovine serum and incubated with test and reference compounds for 24 h before measuring luminescence. Data were fitted by the means of four parameter logistic regression.

### Pharmacokinetic studies

#### Collection of human blood samples

Anonymized human blood samples were obtained from healthy volunteer donors upon informed written consent and in accordance with national legislation.

#### Stability in whole blood

Compounds were incubated in diluted fresh rat, dog, minipig or human whole blood (heparin stabilized, 50% blood and 50% phosphate buffer with 1% bovine serum albumin) for 120 min at 0.1 µM, at 37 °C and the half-lives were determined. Samples were taken at 0, 15, 30, 60 and 120 min, and reactions were terminated by addition of acetonitrile containing analytical internal standard (IS). Test compound depletion was determined using time-of-flight mass spectrometry in positive ion mode (TOF–MS; AB Sciex API5600). Elimination rate constants (k) were calculated from peak areas and the half-lives was determined (t_½_ = ln2/k).

#### Hepatocyte stability

Compounds (1.0 µM) were incubated in duplicates with rat, dog, minipig or human hepatocytes (1.0 million cells/mL) in phosphate buffer, in 96-well plates for 120 min (37 °C). Reactions were stopped by addition of cold acetonitrile, at 0, 10, 30, 60, and 120 min, and dispensed into cold acetonitrile to stop the reactions. Plates were centrifuged for 30 min and samples were analysed using liquid chromatography coupled to a time-of-flight mass spectrometer. Compound depletion over time was used to estimate the elimination rate constant, from which the apparent intrinsic clearance (CLapp), and half-life (t½), were calculated.

#### In vivo pharmacokinetics

The pharmacokinetic profile of LEO 134310 was determined in rat (male Sprague Dawley) and dog (female Beagle) after intravenous (i.v.) injection at 1 mg/kg. LEO 134310 was administered in isotonic cyclodextrin solution at 0.5 mg/mL (0.1% Citrate buffer pH 4, 20% hydroxy-propyl-β-cyclodextrin in 0.2% NaCl). The i.v. dose was given as a bolus in rats and as a slow injection over 2 min in dogs. The dosing volume was 2 mL/kg. Blood was immediately diluted after sampling in an inhibitory cocktail containing acetylcholinesterase inhibitor Dichlorvos [1 mg/mL in an 8 mg/mL K_2_EDTA solution in phosphate buffer (PBS)] and samples were stored at − 80 °C until analysis. Samples were analyzed by LC–MS/MS using an API 5500/API 6500 QTRAP mass spectrometer. Pharmacokinetic parameters were derived from a non-compartment model in Phoenix64 (Pharsight, Certara).

To evaluate allometric scaling, actual clearance in dog and man were extracted from the literature for 6 ester drugs^27–38^.

### Human skin explant studies

NativeSkin models (NS002) were purchased from Genoskin, France. Human skin samples from abdominal reduction surgery were obtained by Genoskin from donors upon written informed consent. Full ethical approval for the study protocol was obtained from the French ethical research committee (Comité de Protection des Personnes), and authorization was given by the French Ministry of Research. The study was conducted according to the Declaration of Helsinki. 8 mm punch biopsies were excised and embedded in a fibrin-based matrix in transwells and a silicone ring was firmly attached to the surface covering the outer edge of the skin biopsy to prevent lateral diffusion of topically applied formulations. Skin models were cultured in a defined hydrocortisone- and serum-free medium provided by Genoskin.

LEO 134310 and reference glucocorticoids were dissolved in propylene carbonate and tested at 4 concentrations in five- or tenfold titrations. Compounds were applied in duplicates or triplicates on the skin surface (0.5 cm^2^) in a volume of 2 µL and incubated for 24 h. Vehicle and untreated samples were included for basal level of expression.

RNA was extracted and qPCR was performed using TaqMan gene expression assays (*FKBP5*-Hs01561006_m1, *TSC22D3 (GILZ)*-Hs00608272_m1, *TXNIP*-Hs01006900_g1, *ACTB*-Hs99999903_m1, *PPIA*-Hs99999904_m1, *GAPDH*-Hs99999905_m1). Gene expression of *FKBP5*,*TSC22D3 (GILZ)*, and *TXNIP* was normalized to the geometric mean of the reference genes *ACTB*, *PPIA* and *GAPDH*.

Doses were expressed as compound (API) concentrations (µg/mL) in the applied formulations. Relative upregulation of *FKBP5* was estimated as − fold to vehicle treated control. Effective doses (E_max_ and ED_50_) were determined from a sigmoid E_max_ model in Phoenix64 version 8.0, Pharsight, Certara. Mean values and standard deviation of E_max_ and ED_50_ were calculated by weighing individual experiments based on the intra- and inter-experiment variability^40^.

### Local tolerance study in minipigs

5 male Göttingen SPF minipigs (5–7 months old; body weight 12–15 kg) were obtained from Ellegaard Göttingen Minipigs A/S, Dalmose, Denmark.

Each animal was treated for 6 h daily over 28 days with 25 µL of the following topical formulations on an application area of 2.5 cm × 2.5 cm: Vehicle (transcutol:acetone (1:9) with 10% hydroxy-propyl-cellulose); Dermovate cream (CP 0.05%), CP 0.05% in vehicle; Betnovate cream (BMV 0.1%); BMV 0.1% in vehicle; LEO 134310 2% in vehicle. One test field on each animal remained untreated.

Formulations were spread uniformly over the indicated clipped area and covered with porous gauze dressing. At the end of each exposure period all application site as well as the untreated area were rinsed with a mild soap (Abena Mild cream soap, Abena A/S, Denmark) in water and dried with a paper towel.

Skin biopsies were taken from anaesthetized animals 24 h after the last treatment. Samples for histopathological examination were fixed in phosphate buffered neutral 4% formaldehyde and stained with H&E. Mean arithmetic epidermal thickness was determined by measuring epidermal area, excluding stratum corneum, and dividing this area with the length of the epidermis. All measurements were performed on H&E stained and scanned slides using the Visiopharm software.

RNA was extracted from the biopsies and qPCR was performed using customized TaqMan gene expression assays for *FKBP5* and *TBP* (reference gene).

### TPA induced skin inflammation in mice

Female Balb/c mice of 7 to 8 weeks old were obtained from Charles River, Germany and acclimatized for at least 1 week. Skin inflammation was induced by repeated topical application of TPA (20 μL of 0.01% TPA in acetone; 10 μL to each side of ear) on days 1, 3, 5, 8 and 10. On day 8, all TPA-treated animals were stratified so that mean ear thickness was comparable for all experimental groups.

Treatments were performed by topical application of either vehicle (90% acetone, 10% transcutol), Treatments were applied twice daily on days 8, 9, 10, and once on day 11 (20–10 μL to each side of ear) with at least 5 h between the first and second treatment. TPA challenge on days 8 and 10 was performed at least 1 h after the first treatment. On day 11, animals were sacrificed 2 h after the last dosing. Blood was sampled by cardiac puncture from animals anaesthetized with isoflurane. Thereafter, ear biopsies were taken with a biopsy punch (8 mm diameter), weighed and stored at – 80 °C. Plasma osteocalcin levels were analyzed by EIA (rat-MID, IDS, UK). Effective inhibitory doses (ID_50_) were determined from a sigmoid I(max) model in Phoenix64, Pharsight, Certara. Therapeutic Index (TI) was calculated either as ID_50_ for spleen weight reduction or ID_50_ for blood osteocalcin concentrations, divided by ID_50_ for ear weight reduction.

RNA was extracted from biopsies and qPCR was performed using TaqMan gene expression assays (*Fkbp5*-Mm00487406_m1, *Tsc22d3 (Gilz)*-Mm00726417_s1, *Actb*-Mm00607939_s1, *Pgk1*-Mm00435617_m1, *Tbp*-Mm00446971_m1, *Ubc*-Mm02525934_g1). Gene expression of *Fkbp5* and *Tsc22d3* (*Gilz*) was normalized to the geometric mean of the reference genes *Actb*, *Pgk1*, *Tbp* and *Ubc*.

### In vivo study procedures

Animal experimental procedures as well as animal housing and husbandry were compliant with Danish legislation (BEK nr 88 30/01/2013), EU Directive 2010/63/EU, and the project license approved by the Danish authorities for animal experimentation (Rådet for Dyreforsøg). The ARRIVE guidelines were considered in the study design and planning phase and followed to the extent possible.

## Supplementary Information

Supplementary Information.

## Data Availability

All data generated or analyzed during this study are included in this published article.
