# Supplementary material for: Characterization of a novel non-steroidal glucocorticoid receptor agonist optimized for topical treatment
Source: Sci Rep. 2022 Jan 27;12:1501. doi: 10.1038/s41598-022-05471-w (PMC8795149; doi:10.1038/s41598-022-05471-w)
Supplement: Supplementary file 1 — Supplementary Information. [file 41598_2022_5471_MOESM1_ESM.docx]

**Characterization of a novel non-steroidal glucocorticoid receptor agonist optimized for topical treatment**

**Stefan Eirefelt^*^, Martin Stahlhut, Naila Svitacheva, Martin A. Carnerup, Joel Mauricio Correa Da Rosa, David Adrian Ewald, Troels T. Marstrand, Mikkel Krogh-Madsen, Georg Dünstl, Kevin Neil Dack, Anna Ollerstam, Hanne Norsgaard**

**LEO Pharma A/S, Industriparken 55, Ballerup, Denmark.s**

***Corresponding author:** [**stefan.eirefelt@leo-pharma.com**](mailto:stefan.eirefelt@leo-pharma.com)

**Supplementary Methods**

*Mineral corticoid receptor (MR) agonist and antagonist data (Figure S1).*

LEO 134310 and LEO 134988 were tested in the human Mineralocorticoid Receptor in cellular GAL4 reporter gene assays. All assays were done in HEK293 cells (DSMZ ACC 305). The plasmids used in Phenex’ GAL4 assay system are derivatives of Stratagene’s M2H plasmids: the reporter plasmid pFR-Luc (contains a synthetic promoter with five tandem repeats of the yeast GAL4 binding sites that control expression of the Photinus pyralis (American firefly) luciferase gene), and pCMV-BD (for fusions of nuclear receptor ligand binding domains to the DNA-binding domain of the yeast protein GAL4). In order to improve experimental accuracy, a second reporter - Renilla reniformis luciferase, driven by a constitutive promoter - was included as internal control. Using the control reporter (pRL-CMV) allows correcting for variations in experimental handling e.g. transfection efficacy, cell viability, pipetting errors, cell lysis efficiency and assay efficiency. The compounds were tested in 9 concentrations in triplicates.

*Selectivity against nine different nuclear hormone receptors (Figure S2).*

Selectivity screening on nuclear receptors – Studies were performed at CEREP using their proprietary assays for human PPARalpha, LXRbeta, RARalpha, RXRalpha, ERalpha, ERbeta, progesterone receptor, androgen receptor and thyroid hormone receptor employing specific, radiolabeled agonist ligands. Interactions leading to greater than 25% displacement of the radiolabeled agonist ligand were considered significant. Concentrations of LEO 134310 and LEO 134998 were 10µM.

*Reversal of GR agonist-mediated suppression of cytokine release from LPS-stimulated PBMC by RU846 (Figure S3).*

PMBCs were isolated from human buffy coats obtained in accordance with national legislation and following in-house protocol at Charles River Laboratories (Leiden, Netherlands). Test compounds and PBMCs were incubated with 10 ng/ml LPS (Sigma) overnight at 37°C in HEPES and Glutamax-containing RPMI medium supplemented with 0.5% human serum albumin (human PBMCs) and penicillin/streptomycin. TNF-alpha, IL-1-beta, and IL-6 were measured by MSD multiplex assay (Meso Scale Discovery).*Compound concentration in dermis (Figure S4)*

The concentrations of the compounds in dermis were determined from skin biopsies from the human skin explants. After removal of SC by tape stripping, the skin biopsies were separated into dermis and epidermis using an ultra-clean tweezers. Subsequently, the dermis biopsies were homogenized in 400 µL of citrate buffer pH 4.0:acetonitrile (50:50) in 2 mL BeadRuptor vials containing ceramic beads, applying 5 cycles of homogenization of each 20 s in a BeadRuptor™ (Omni International). After homogenization, the samples were diluted, centrifuged and precipitated with acetonitrile containing internal standard (IS), and the compounds were thereafter quantified by means of liquid chromatography-tandem mass spectrometry (LC-MS/MS) using an AB Sciex 5500 or 6500 QTRAP. Calibration curves were prepared by spiking the analytes in blank homogenate, and a generic IS were used in quantification. The dermis micro molar concentrations were calculated assuming 1 gram of skin tissue equal to 1 mL.

**Supplementary Figures and Tables**


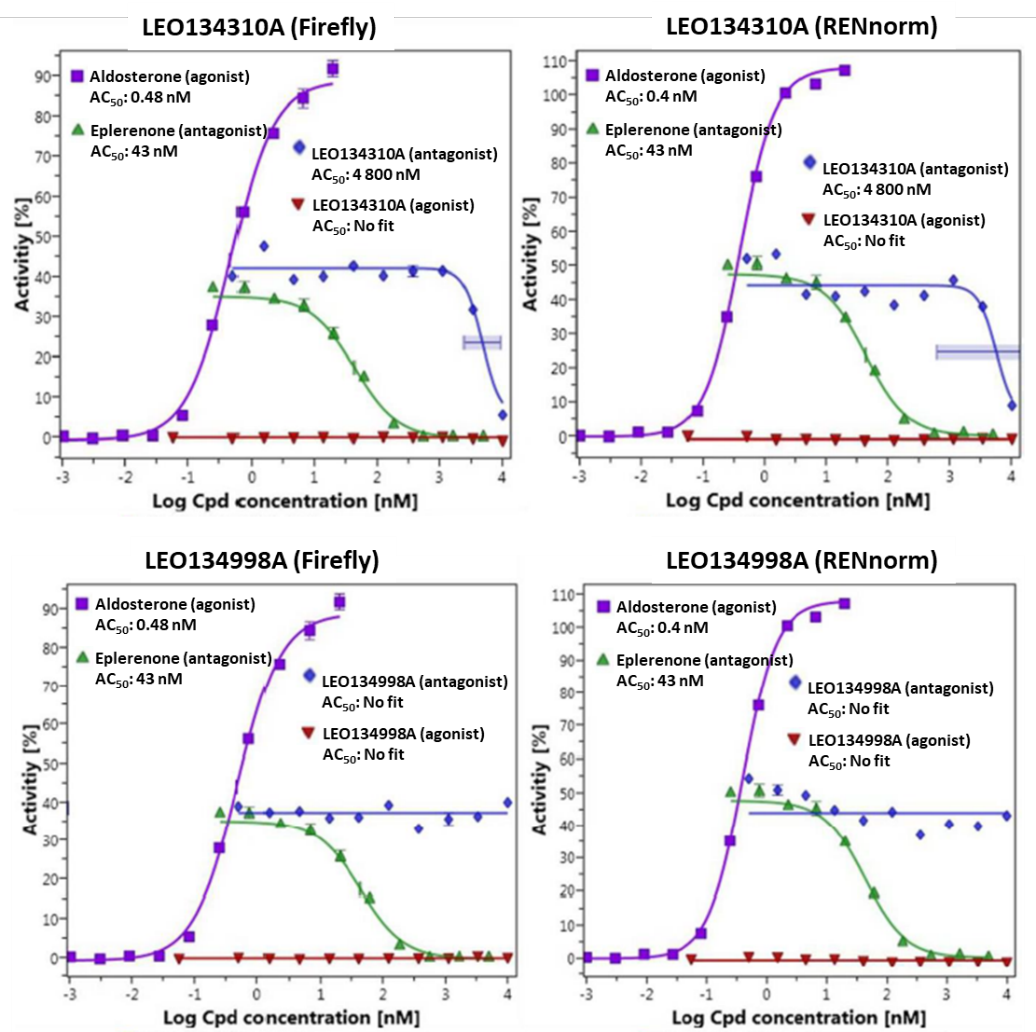


**Figure S1.** Percent luciferase activity after MR stimulation with LEO134310A and LEO134998A) in a mammalian-1-hybrid set up (cellular GAL4 reporter gene assays). Aldosterone and eplerenone were used as agonist and antagonist control, respectively. Left panels: MR activity compared to aldosterone and eplerenone. Right panels: MR activity normalized to constitutive Renilla luciferase expression.

1. **LEO 134310**


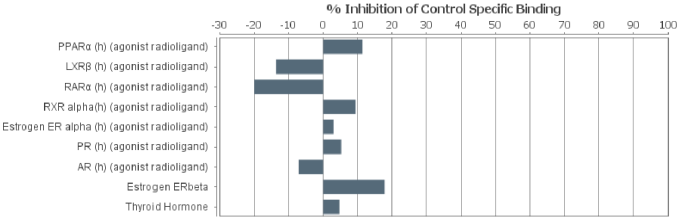


1. **LEO 134998**


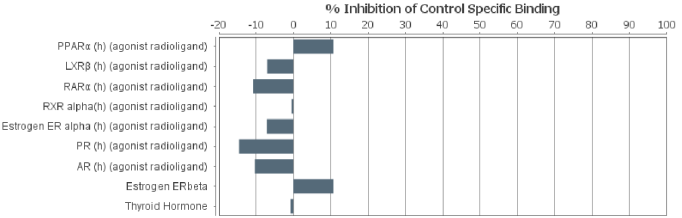


**Figure S2.** Inhibition (%) of control specific binding for LEO134310A (A) and LEO134998A (B)


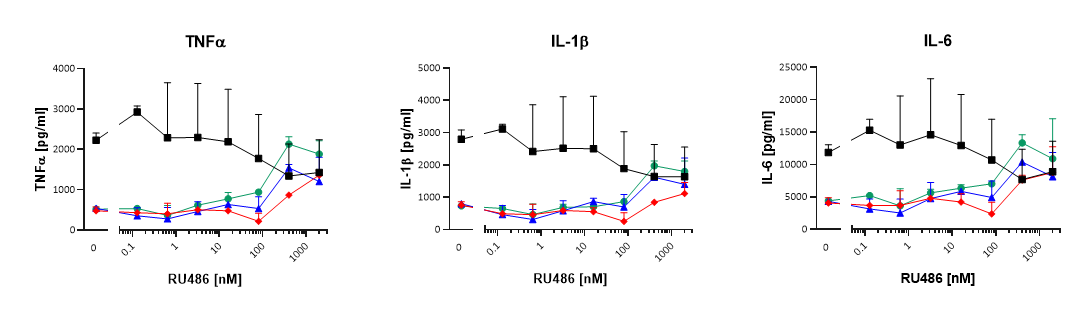


**Figure S3.** Concentration-dependent reversal of GR agonist-mediated inhibition of TNF-alpha (left panel), IL-1-beta (middle panel) and IL-6 (right panel) by the GR antagonist RU486. Vehicle (■), 100 nM CP (♦), 100 nM BMV (▲), 100 nM LEO 134310 (●).


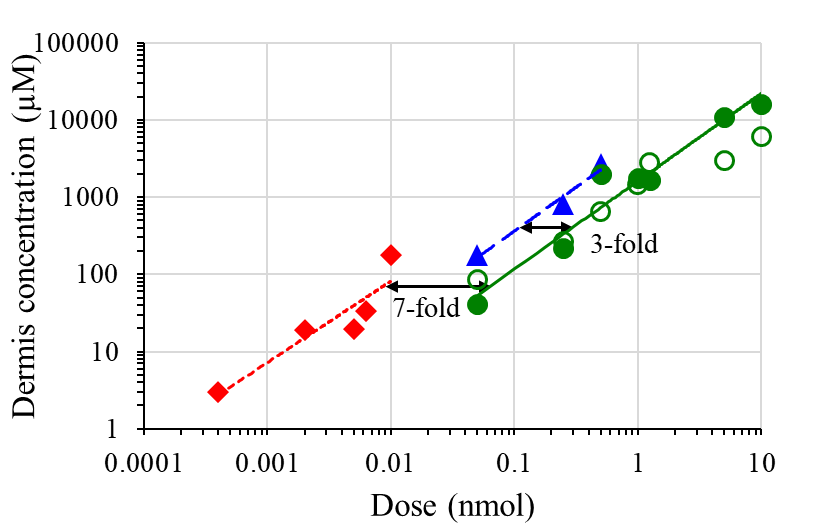


**Figure S4.** Dermis concentration vs. dose, in human skin explant at 24h after dosing solutions in propylene carbonate. Mean values (n=1-5 for each dose). Trend line shown for parent compounds. CP (♦), BMV (▲), LEO 134310 (●) and LEO 134988 (after dosing LEO 134310) (○).


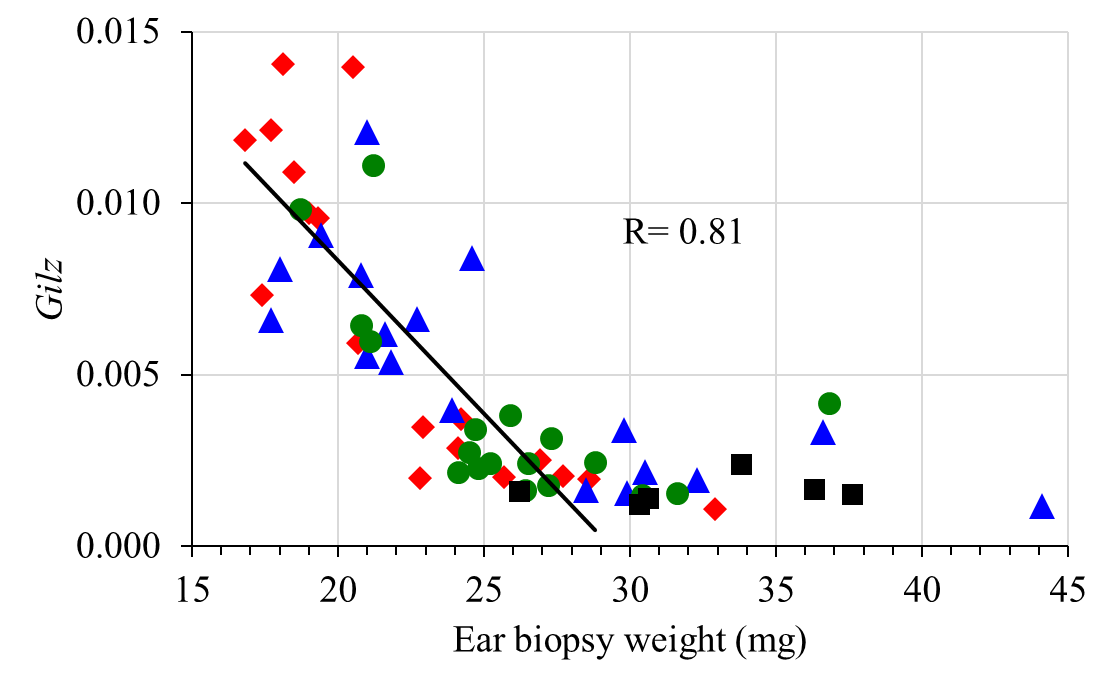


**Figure S5.** mRNA levels of *Gilz* vs ear biopsy weight (R= correlation coefficient). Vehicle (■), CP (♦), BMV (▲), LEO 134310 (●).


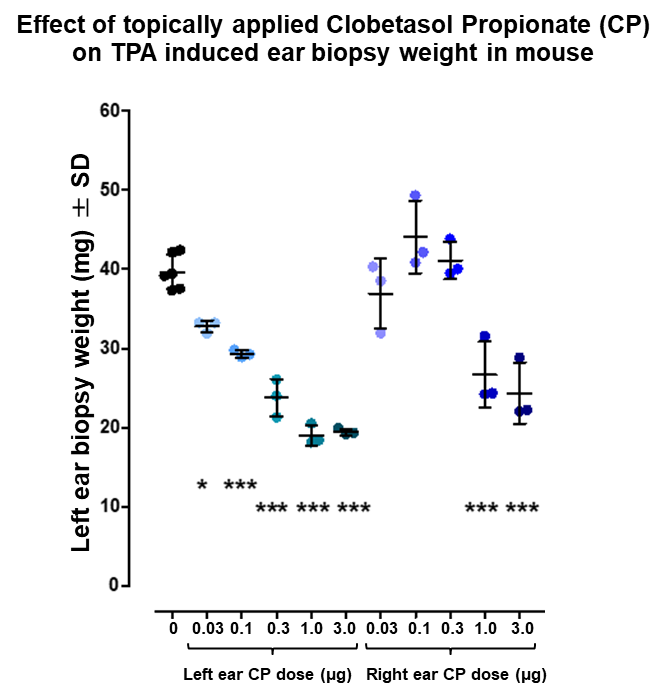

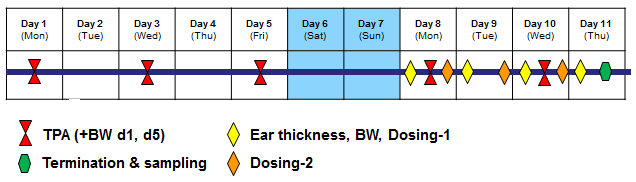


**Figure S6.** Effect of topically applied CP on TPA induced ear biopsy weight in mice (upper panel). Treatment scheme can be seen in the lower panel. Ear oedema was induced by TPA on the left ear on day 1, 3, 5, 8 and 10 and CP was topically administered daily on either left or right ear on day 8 to 11. The left ear biopsy weight was measured on day 11. Significant effect was seen also when CP was administered on the right ear at the 2 highest doses, indicating contribution to the local effect, by systemic exposure

**Table S1.** Effective doses (API concentrations in dosing solution) of CP (n= 6 studies), BMV (n= 3 studies) and LEO134310A (n= 5 studies) to induce *GILZ* or *TXNIP* levels to 50% of maximum response (ED_50_). Gene expression levels were normalised to vehicle-treated controls and E_max_ and ED_50_ were estimated in a sigmoid E_max_ model. Weighted geometric mean ± standard deviation. Statistical comparison (T-test) against BMV (NS= Not Significant).

| **Compound** | ***GILZ*** | | ***TXNIP*** | |
| --- | --- | --- | --- | --- |
|  | **Relative ED_50_**  **[µg/mL]** | **Emax**  **[fold to control]** | **Relative ED_50_**  **[µg/mL]** | **Emax**  **[fold to control]** |
| CP | 1.1 ± 0.25^NS^ | 7.7 ± 2.5^NS^ | 1.3 ± 0.33^***^ | 19 ± 8.5^NS^ |
| BMV | 5.1 ± 27 | 6.1 ± 3.7 | 52 ± 15 | 17 ± 17 |
| LEO 134310 | 270 ± 110^**^ | 6.7 ± 2.7^NS^ | 110 ± 300^NS^ | 19 ± 7.3^NS^ |
